# An observational comparative study of Hass avocado fruit quality and its associations with rhizosphere soil properties across four representative orchards in China

**DOI:** 10.3389/fpls.2026.1890031

**Published:** 2026-08-21

**Authors:** Changxiang Yang, Liangyi Zhao, Xibin Zhang, Chengping Luo, Tingfa Wang, Baoqiong Zhang, Xuehu Yang, Zekun Shi, Weifeng Zhao, Jin Xu, Liangliang Sun

**Affiliations:** 1 College of Tropical Crop, Yunnan Agricultural University, Kunming, China; 2 International Joint Laboratory for Green Production Technology of Special Fruits and Vegetables in Yunnan Province, Yunnan Agricultural University, Kunming, China; 3 Avocado Science and Technology Research Centre of Menglian County, Puer, China; 4 College of Horticulture, Shanxi Agricultural University, Taiyuan, China

**Keywords:** avocado, correlation analysis, production optimization, quality analysis, quality formation, soil characteristics

## Abstract

**Introduction:**

Avocado (Persea americana Mill.) quality is significantly influenced by soil conditions, as well as climatic factors, fertilization regimes, and orchard management practices. However, systematic research on the combined effects of these factors for 'Hass' in China remains limited.

**Methods:**

This observational comparative study compared four producing regions, Menglian (ML), Yuanmou (YM), Xishuangbanna (XSBN), and Longzhou (LZ), by analyzing fruit quality, rhizosphere soil properties, enzyme activities, and mineral elements using standard analytical procedures and multivariate analysis.

**Results:**

The results showed distinct regional characteristics: ML fruits were pear-shaped and had the highest edible yield (77.91%); YM produced the largest single fruit weight (194.36 g) and thickest pulp (2.38 cm); XSBN excelled in vitamin C (38.87 mg/100 g) and soluble sugars (4.91%); LZ showed the highest K (6.91 mg/kg) and Ca (81.51 mg/kg). Soil properties also varied significantly. ML soil had the highest Fe, Mn, Cu, and catalase activity; YM soil had the highest pH, K, and alkaline phosphatase; XSBN soil was richest in organic matter and total N; and LZ soil had the highest P and As.

**Discussion:**

Exploratory correlation analysis revealed that soil enzyme activities and elements such as Mg, Ca, K, and Mn showed strong associations with fruit size, sugar-acid balance, and nutritional composition. These findings identify key soil factors correlated with avocado quality and provide an exploratory basis for region-specific soil management aimed at production optimization. The regional variation in fruit quality highlights the added value of Chinese-grown Hass avocado as a distinctive regional product.

## 1 Introduction

Avocado (*Persea americana* Mill.), also known as alligator pear or butter pear, is an evergreen tree of the genus Persea in the Lauraceae family (Chen et al., 2009). Its fruit is a crop of significant economic value with increasing commercial importance in China. Avocado fruits are rich in carbohydrates, proteins, unsaturated fatty acids, vitamins (A, B, C, D, E, K), and minerals (Dreher and Davenport, 2013; Galvão et al., 2014; Arackal and Parameshwari, 2021). The fruit also contains high levels of phenolic compounds and flavonoids, which contribute to its nutritional quality and antioxidant capacity (Dibacto et al., 2021; Bhuyan et al., 2019). Notably, the accumulation of these bioactive compounds in avocado fruit is strongly influenced by soil nutrient availability, particularly mineral elements such as Mg, Mn, and Zn, which serve as cofactors for key enzymes in secondary metabolic pathways (Hänsch and Mendel, 2009; Kováčik et al., 2010). Overall, avocado represents a high-quality nutritional source that combines premium fats, abundant micronutrients, and high bioavailability (Yang et al., 2024), making it a valuable raw material for the food industry.

Avocado has strong adaptability to soil types and can grow in sandy soil to clay and other soils. The ideal soil depth for root development and nutrient absorption is usually 0.8-1.0 m, and the suitable pH range is 5.5-6.5 (Mejía-Bonilla et al., 2025). Soil properties fundamentally influence avocado growth and fruit quality formation through multiple interconnected mechanisms. Soil pH directly regulates nutrient availability by affecting the solubility and plant uptake of essential elements; for example, micronutrients such as Fe, Mn, and Zn become less available in alkaline soils, while Ca and Mg availability is influenced by soil cation exchange capacity (Bonomelli et al., 2019). Soil organic matter content affects soil structure, water retention, and the supply of nitrogen and other nutrients through mineralization processes (Silber et al., 2018). Furthermore, soil enzyme activities, particularly alkaline phosphatase, sucrase, and urease, mediate the transformation of organic nutrients into plant-available forms, thereby influencing nutrient cycling and plant uptake efficiency (Sinsabaugh et al., 2008). The composition of mineral elements in the rhizosphere, including macro-elements (N, P, K, Ca, Mg) and micro-elements (Fe, Mn, Cu, Zn), directly affects tree nutrition, photosynthetic efficiency, and the subsequent allocation of assimilates to fruit development (Reddy et al., 2014). These soil-plant interactions collectively determine fruit physical traits (size, shape, pulp thickness), nutritional composition (fat, sugar, vitamin content), and mineral element accumulation in the fruit.

Previous cross-regional comparative studies have confirmed that soil conditions significantly influence avocado fruit quality. For example, a study in Tanzania showed significant differences in morphological indicators including longitudinal diameter, transverse diameter, and pulp thickness under different soil pH and altitude conditions, revealing correlations between soil pH and fruit characteristics (Yangaza et al., 2024). In Mexico, difference in soil fertility between the two orchards significantly affected leaf mineral element content. Nutritional diagnosis revealed that one orchard lacked nitrogen (N), Zn, Mn, Fe, and boron (B), while the other was deficient in P, K, Ca, Mn, Zn, and sulfur (S), providing a basis for fertilization management (Sotelo-Nava et al., 2017). In Colombia, studies have demonstrated that soil type influences calcium absorption and partitioning in avocado trees, with implications for fruit quality and postharvest performance (Bonomelli et al., 2019). Recent studies have further demonstrated that the multidimensional quality (including physical, chemical, and sensory characteristics) of Hass avocados is determined by the complex interactions among soil properties, climatic conditions, and harvesting seasonality (López-Hoyos et al., 2024). Specifically, research has shown that soil pH, organic matter content, and nutrient availability interact with climatic variables such as temperature and precipitation to influence fruit yield, size, and nutritional composition (López-Hoyos et al., 2024; Ullah and Joyce, 2024). In addition, the study pointed out that climate factors (such as temperature, precipitation) and soil characteristics (such as pH, organic matter) jointly affect the yield and quality of avocado. These findings collectively emphasize that soil and climate are key environmental variables affecting avocado yield and quality, and that understanding their interactions is essential for optimizing production. These studies emphasize that soil properties, including pH, organic matter content, nutrient availability, and enzyme activities, are key determinants of avocado yield and quality. However, the specific soil characteristics that most strongly influence fruit quality traits, and the relative importance of different soil parameters, remain unclear, particularly in the context of Chinese production systems. Therefore, understanding the relationship between soil properties and fruit quality across different production regions is important for guiding regional cultivation practices and achieving consistent product quality.

Soil properties are the basis of avocado growth and development and fruit formation (Bonomelli et al., 2019). Specifically, soil organic matter and pH directly regulate nutrient availability and significantly affect plant nutrient uptake. Among various soil fertility factors, the supply of N, P and K is particularly critical for avocado fruit yield and quality. Therefore, scientific soil management is a necessary condition for achieving high yield and quality of avocado (Silber et al., 2018). Among global avocado cultivars, Hass is the most representative variety due to its excellent commodity properties and adaptability (García et al., 2021). However, despite its importance, systematic research on the relationship between Hass avocado fruit quality, particularly physical and nutritional properties, and soil characteristics across China’s major production areas remains insufficient. This knowledge gap restricts the development of targeted soil management strategies to optimize fruit quality.

Despite the recognized importance of soil factors in avocado production, three knowledge gaps remain unresolved. First, it is unclear which specific soil properties, including physicochemical parameters, enzyme activities, and mineral elements, are most strongly associated with fruit quality traits (Bonomelli et al., 2019; Ullah and Joyce, 2024). Second, the relative associations of soil enzyme activities versus mineral elements with fruit quality have not been comparatively explored (Sinsabaugh et al., 2008; Reddy et al., 2014). Third, whether distinct, region-specific fruit quality profiles exist across major Chinese production areas remains unexplored (Méndez Hernández et al., 2024). To address these gaps, this study was designed to answer: (i) Which soil properties are primarily associated with Hass avocado fruit quality? (ii) Do soil enzyme activities or mineral elements show stronger associations with key fruit quality traits? (iii) Do distinct quality profiles exist among the four production regions?

By establishing empirical links between soil characteristics and avocado quality, this study aims to generate practical knowledge for soil-based production optimization. Therefore, this study focused on the Hass avocado and rhizosphere soil from four typical production areas in China, Yuanmou (YM), Menglian (ML), Xishuangbanna (XSBN) and Longzhou (LZ). The aims were: (1) to analyze the fruit physical characteristics, nutritional components, and mineral element content; (2) to characterize rhizosphere soil properties, including physicochemical properties, enzyme activities, and mineral elements; (3) to exploratorily examine relationships between fruit quality and soil characteristics using correlation analysis and PCA, identifying primary soil factors associated with regional quality differentiation. The ultimate goal is to provide an exploratory scientific basis for region-specific soil management that supports sustainable avocado cultivation. It is important to emphasize that this study is observational and correlational in design; therefore, causal relationships cannot be inferred from the results. The findings should be interpreted as generating hypotheses for future experimental validation rather than establishing causal effects.

## 2 Materials and methods

### 2.1 Experimental materials and site description

This study selected four major avocado producing regions in China, including three orchards in Yunnan Province (Menglian (ML), Yuanmou (YM) and Xishuangbanna (XSBN)) and one orchard in Guangxi Zhuang Autonomous Region (Longzhou (LZ)). The four orchards selected for this study are all representative orchards within their respective production areas. The selection of these orchards was based on the following considerations: (i) they are the earliest and largest commercial Hass avocado plantations in their respective regions; (ii) they adopt standard cultivation methods that conform to local typical characteristics; (iii) they have been established as local demonstration orchards. The experimental samples were collected from the above orchards, and the climatic conditions in each production area were significantly different. The specific data regarding the average annual rainfall, sunshine duration and temperature in the avocado plantation are presented in **Supplementary Table 1**. The basic topographic characteristics (slope aspect, slope and altitude) and soil types of each sampling point are also summarized in **Supplementary Table 1**. All the sampled fruit trees were Hass varieties, with a tree age of 7–8 years, from certified nurseries. The Hass avocado trees (7–8 years old) in these orchards are all in the main commercial production stage in each region. During the sampling process, we visited farmers, recorded and compared their irrigation, fertilization and other agricultural management measures. **Supplementary Table 2** provides detailed information on irrigation and fertilization regimes for each orchard, as these practices may influence fruit quality and should be considered when explaining regional differences. It should be noted that in this study, each production area only contained one orchard. Due to the differences in climate (temperature, rainfall, sunshine duration), management history, irrigation methods and fertilization systems among different regions (as detailed in **Supplementary Tables 1**, **S2**), these confounding factors represent a significant limitation of the study design. The four orchards differ not only in soil properties but also in irrigation methods (drip vs. flood vs. sprinkler), fertilization rates, application frequencies, and additional practices such as mulching and liming (**Supplementary Table 2**). These management practices could substantially influence both soil characteristics and fruit quality, and they vary simultaneously with soil properties across regions. Therefore, the observed differences in fruit quality cannot be attributed solely to soil properties. Rather, this study should be viewed as an observational comparative study that identifies associations between soil characteristics and fruit quality, generating hypotheses for future controlled investigations.

Fruit and soil samples were collected simultaneously from all four production areas on the same day. Within each region, three sampling locations (spatially separated within the orchard, at least 50 m apart) were randomly selected. At each location, three healthy avocado trees of similar size and vigor were chosen as sample trees, resulting in a total of 9 trees per region (3 locations × 3 trees). The individual tree was considered the experimental unit for all statistical analyses (n=3 biological replicates per region, as data from the three trees at each location were averaged to obtain a representative value for that location, and then the three locations were treated as independent replicates). From each tree, ten fruits meeting the following criteria were collected: typical Hass shape, consistent medium size, maturity indicated by slightly softened pulp, and absence of disease, insect damage, or mechanical injury. The 10 fruits from each tree were pooled and homogenized for subsequent analyses to obtain a representative value for that tree. Thus, for each region, fruit quality data were derived from three biological replicates (trees), with all measurements performed in triplicate for each replicate. The n=3 value (representing three trees per region) applies to all figures and tables presented in this study, unless otherwise specified. We used soil corers to collect rhizosphere soil samples from a depth of 5–40 cm in the soil layer 2 m below the tree trunks of each tree. A total of 36 soil samples were collected (4 regions × 3 locations × 3 trees). For each tree, soil samples were collected from three points around the tree trunk and pooled to form a composite sample for that tree. Thus, soil data were also derived from three biological replicates (trees) per region, consistent with the fruit sampling design. Soil samples were divided into two portions: one portion was air-dried in a ventilated area, ground, and passed through a 100-mesh sieve for subsequent chemical analysis; the other portion was stored at -80 °C for the determination of soil enzyme activities.

### 2.2 Analysis of soil properties

Soil physical and chemical properties were determined using standard analytical methods. Soil pH was measured in a 1:2.5 (w/v) soil-to-water suspension using a Mettler Toledo FE28 pH meter (Mettler Toledo, Switzerland). Soil organic matter (SOM) content was determined by the potassium dichromate oxidation method (Walkley-Black). Total nitrogen (TN) was analyzed using the Kjeldahl method with a Kjeltec 8400 auto-analyzer (FOSS, Denmark). Alkali-hydrolyzable nitrogen (AN) was determined by the alkaline hydrolysis diffusion method. Total phosphorus (TP) and available phosphorus (AP) were measured by the molybdenum antimony colorimetric method using a Shimadzu UV-2600 spectrophotometer (Shimadzu, Japan) after digestion with HClO_4_-H_2_SO_4_ and extraction with NaHCO_3_ (Olsen method), respectively. Total potassium (TK) and available potassium (AK) were analyzed by flame photometry using a Sherwood M410 flame photometer (Sherwood Scientific, UK), following digestion with HF-HClO_4_ and extraction with NH_4_OAc, respectively.

The contents of mineral elements in the soil, including calcium (Ca), magnesium (Mg), iron (Fe), manganese (Mn), copper (Cu), and zinc (Zn), were determined by inductively coupled plasma optical emission spectrometry (ICP-OES, Agilent 5110, Agilent Technologies, USA) after digestion with HNO_3_-HF-HClO_4_. Calibration was performed using multi-element standard solutions (AccuStandard, USA) with concentrations ranging from 0 to 50 mg/L. The detection limits for soil mineral elements were as follows: Ca: 0.05 g/kg, Mg: 0.02 g/kg, Fe: 0.5 mg/kg, Mn: 0.1 mg/kg, Cu: 0.05 mg/kg, and Zn: 0.1 mg/kg. Quality control was ensured using certified reference materials (GSS series, National Research Center for Certified Reference Materials, China), reagent blanks, and duplicate analyses (one duplicate per 10 samples). Recovery rates for all elements ranged from 92% to 105%. The contents of heavy metals cadmium (Cd) and arsenic (As) were analyzed by atomic fluorescence spectrometry (AFS-9700, Beijing Haiguang Instrument Co., China) following appropriate digestion procedures. The detection limits for Cd and As were 0.002 mg/kg and 0.005 mg/kg, respectively. Certified reference materials (GSS series) were used for quality control, with recovery rates of 90-108% for Cd and 88-105% for As.

Soil enzyme activities were assessed using colorimetric methods with a Shimadzu UV-2600 spectrophotometer (Shimadzu, Japan). Catalase (CAT) activity was measured by monitoring the decomposition of H_2_O_2_. Sucrase activity was determined based on the production of reducing sugars from sucrose. Alkaline phosphatase (ALP) activity was assessed using disodium phenyl phosphate as a substrate. Urease activity was measured by quantifying the ammonium nitrogen released from urea hydrolysis (Yang et al., 2025). All enzyme assays were performed in triplicate, and reagent blanks were included for each assay. Calibration curves were constructed using appropriate standards (glucose for sucrase, phenol for ALP, and NH_4_Cl for urease) with correlation coefficients (R²) > 0.995. Quality control was ensured through the inclusion of a standard soil sample in each batch of analysis.

### 2.3 The determination method of the apparent morphological indicators of avocado fruits

Single fruit weight was measured using an analytical balance. Fruit longitudinal diameter, fruit transverse diameter, seed longitudinal diameter, and seed transverse diameter were measured using a vernier caliper. Pulp thickness was measured using a ruler. The fruit shape index was calculated as the fruit longitudinal diameter divided by the fruit transverse diameter:

Fruit Shape Index = Fruit Longitudinal Diameter/Fruit Transverse Diameter

The seed shape index was calculated as the seed longitudinal diameter divided by the seed transverse diameter:

Seed Shape Index = Seed Longitudinal Diameter/Seed Transverse Diameter

The fruit edible portion yield was calculated as the single fruit weight minus the mass of the peel and seed, divided by the whole fruit weight:

Fruit Edible Portion Yield = [(Fruit Weight - (Seed Mass + Peel Mass))/Fruit Weight] × 100%

### 2.4 The determination method of crude fat content in avocado fruits

Crude Fat Content was determined using the Soxhlet extraction method with a Soxhlet apparatus (B-811, Buchi, Switzerland). Five g (accurate to 0.0001g) of avocado fruits pulp was weighed into a sealed glass container. An appropriate amount of sea sand was added, and the mixture was placed in a boiling water bath to evaporate moisture. After removing the moisture, the sample was placed into a Soxhlet extractor cartridge. Petroleum ether (boiling point 60-90 °C, Sinopharm Chemical Reagent Co., China) was added for reflux extraction. Following extraction, the solvent was recovered and evaporated to dryness using a rotary evaporator (Buchi R-300, Switzerland). The residue was dried at 105 °C to constant weight and weighed. This was repeated (typically three times) until no more fat could be extracted. All extracts were combined. Quality control was performed using a standard oil sample (Soybean oil certified reference material, GBW(E)10015, China) with each batch of extractions.

The fat content was calculated using the formula:

X = [(m_1_ - m_2_)/m] × 100
X = Fat content (%)
m_1_ = Mass of the flask and crude fat (g)
m_2_ = Mass of the flask (g)
m = Mass of the sample (g)

### 2.5 Method for determining the content of vitamin C

Vitamin C content was determined using the 2,6-dichlorophenolindophenol method (AOAC Official Method 967.21). A 2.5 g sample of avocado pulp (accurate to 0.0001g) was placed in a mortar and ground to a paste under ice-bath conditions. The homogenate was diluted to a final volume of 25 mL with a 2% oxalic acid solution. A 10 mL aliquot of the filtrate was pipetted into a conical flask and titrated with a standardized 2,6-dichlorophenolindophenol solution (prepared fresh daily and standardized against standard ascorbic acid solution) until a faint pink color persisted for at least 15 seconds. The volume of dye used was recorded. A blank titration was performed using 10 mL of 2% oxalic acid solution following the same method. The procedure was repeated three times. The vitamin C content was calculated using a standard calibration curve prepared from L-ascorbic acid (Sigma-Aldrich, USA) at concentrations ranging from 0 to 100 µg/mL. Quality control was ensured by analyzing a certified reference material for vitamin C content (NIST SRM 3280, National Institute of Standards and Technology, USA) with each batch of samples. Recovery rates for spiked samples ranged from 95% to 103%.

### 2.6 Method for determining the content of soluble proteins

Soluble protein content was determined using the Bradford method. Three fruits were randomly selected from the ten fruits harvested from each tree. A 1.0 g sample of finely chopped avocado pulp (accurate to 0.0001g) was homogenized with 8 mL of distilled water in an ice bath. The homogenate was centrifuged at 10,000 r/min for 40 min at 4 °C. The resulting supernatant, representing the soluble protein extract, was collected and stored at low temperature for subsequent analysis using the Bradford method.

### 2.7 Method for determining total flavonoid content

Total flavonoid content was determined using the aluminum chloride colorimetric method (Shraim et al., 2021). A 2.0 g sample of avocado (accurate to 0.0001g) was placed in a 150 mL conical flask, and 30 mL of 75% ethanol was added. After ultrasonic extraction for 1 hour at 40 °C using an ultrasonic bath (KQ-500DE, Kunshan Ultrasonic Instruments Co., China), the mixture was filtered into a 50 mL volumetric flask. The filter paper was rinsed twice with 75% ethanol, and the volume was made up to the mark with the same solvent. A 2.0 mL aliquot of this solution was transferred to a 25 mL volumetric flask. Then, 1.0 mL of a 5% NaNO_2_ solution was added, mixed well, and allowed to stand for 6 min. Subsequently, 1.0 mL of a 10% Al(NO_3_)_3_ solution was added, mixed, and allowed to stand for another 6 min. Finally, 1.0 mL of a 40% NaOH solution was added and the mixture was allowed to stand for 15 min. The absorbance was measured at a wavelength of 510 nm using a Shimadzu UV-2600 spectrophotometer (Shimadzu, Japan). Calibration was performed using rutin standard (Sigma-Aldrich, USA) at concentrations ranging from 10 to 200 µg/mL (R² > 0.999). The detection limit for total flavonoids was 1.5 µg/mL. Quality control was ensured through analysis of a reference standard with each batch of samples and recovery tests by spiking samples with known amounts of rutin standard (recovery rates: 93-104%).

### 2.8 Method for determining the content of mineral elements

Mineral element content was determined using atomic absorption spectrophotometry (AAS, PerkinElmer PinAAcle 900T, PerkinElmer, USA) equipped with a graphite furnace and flame atomizer. Three fruits were selected from each production area. A 0.2 g sample of dried and ground avocado pulp was weighed and digested with 5 mL of concentrated HNO_3_ (ultra-pure grade) and 2 mL of H_2_O_2_ (30%) using a microwave digestion system (MARS 6, CEM Corporation, USA). The digested solution was diluted to 10 mL with ultra-pure water and analyzed by AAS. Calibration standards were prepared from multi-element standard solutions (AccuStandard, USA) for each element, with concentrations ranging from 0.01 to 10 mg/L depending on the element. The detection limits were: Fe: 0.01 mg/kg, Mn: 0.002 mg/kg, Cu: 0.001 mg/kg, Zn: 0.005 mg/kg, Ca: 0.5 mg/kg, Mg: 0.05 mg/kg, K: 0.5 mg/kg, and P: 0.5 mg/kg. Quality control was performed using certified reference materials (NIST SRM 1515 Apple Leaves, National Institute of Standards and Technology, USA) and reagent blanks. Recovery rates for all elements ranged from 95% to 107%. All analyses were performed in triplicate.

### 2.9 Principal component analysis methodology

Principal Component Analysis (PCA) was performed to visualize multivariate patterns of fruit quality differentiation among the four production regions. All 28 fruit quality variables (F1–F28) were included without prior selection and standardized using z-score transformation (mean = 0, SD = 1) to ensure equal contribution across different units and scales. Principal components were retained based on the Kaiser criterion (eigenvalues > 1), and cumulative explained variance was calculated for the retained components. For each component, loading values (eigenvectors) were computed, with variables having loadings > 0.40 considered meaningful contributors to that component. Confidence ellipses in the PCA score plot represent 95% confidence intervals for regional means (n=3 biological replicates per region, with the tree as the experimental unit), providing a visual indication of multivariate separation among regions.

### 2.10 Data analysis

Data organization and preliminary calculations were conducted using Microsoft Excel 2016. Statistical analysis, specifically one-way analysis of variance (ANOVA), was performed to assess significant differences in fruit quality indicators and soil characteristics using SPSS 26 (IBM Corp., USA). Prior to ANOVA, the assumptions of normality and homogeneity of variances were tested using the Shapiro-Wilk test and Levene’s test, respectively. For all ANOVA analyses, the individual tree served as the experimental unit, with n=3 biological replicates per region (representing three trees). Data from the three locations within each region were averaged per tree before statistical analysis to avoid pseudoreplication. Graphical representations of the data were generated with GraphPad Prism 10.12. All values in the figures and tables are presented as mean ± standard deviation (SD), with n=3 per region. Due to the limitations of the research design (only one orchard in each region, a total of 4 regions), we employed ANOVA to detect regional differences, PCA to visualize the multivariate patterns of fruit quality differences, and paired Pearson or Spearman correlation analysis to explore the correlations between soil and fruit parameters as appropriate exploratory methods. These statistical tools are suitable for generating hypotheses and identifying potential predictors in small-scale observational studies, but they cannot determine causal relationships or quantify the relative contributions of each factor. Given that more than 40 fruit and soil variables were analyzed simultaneously, resulting in a large number of pairwise correlations, we applied the False Discovery Rate (FDR) correction (Benjamini-Hochberg method) to control for the risk of false positives due to multiple comparisons. Correlations with an FDR-adjusted q-value < 0.05 were considered statistically significant. The significance levels reported in the results (P < 0.05, P < 0.01, P < 0.001) correspond to the raw P-values prior to FDR adjustment; all correlations reported as significant had FDR-adjusted q-values < 0.05. Furthermore, correlation analyses between fruit quality parameters and soil properties were visualized using heatmaps constructed with the Genedenovo online heatmap tool. For all correlation analyses, the mean values from each tree (n=3 per region) were used, with the tree as the experimental unit.

## 3 Results and discussions

### 3.1 Fruit physical properties from different avocado producing areas and their analysis

**Figure 1:**
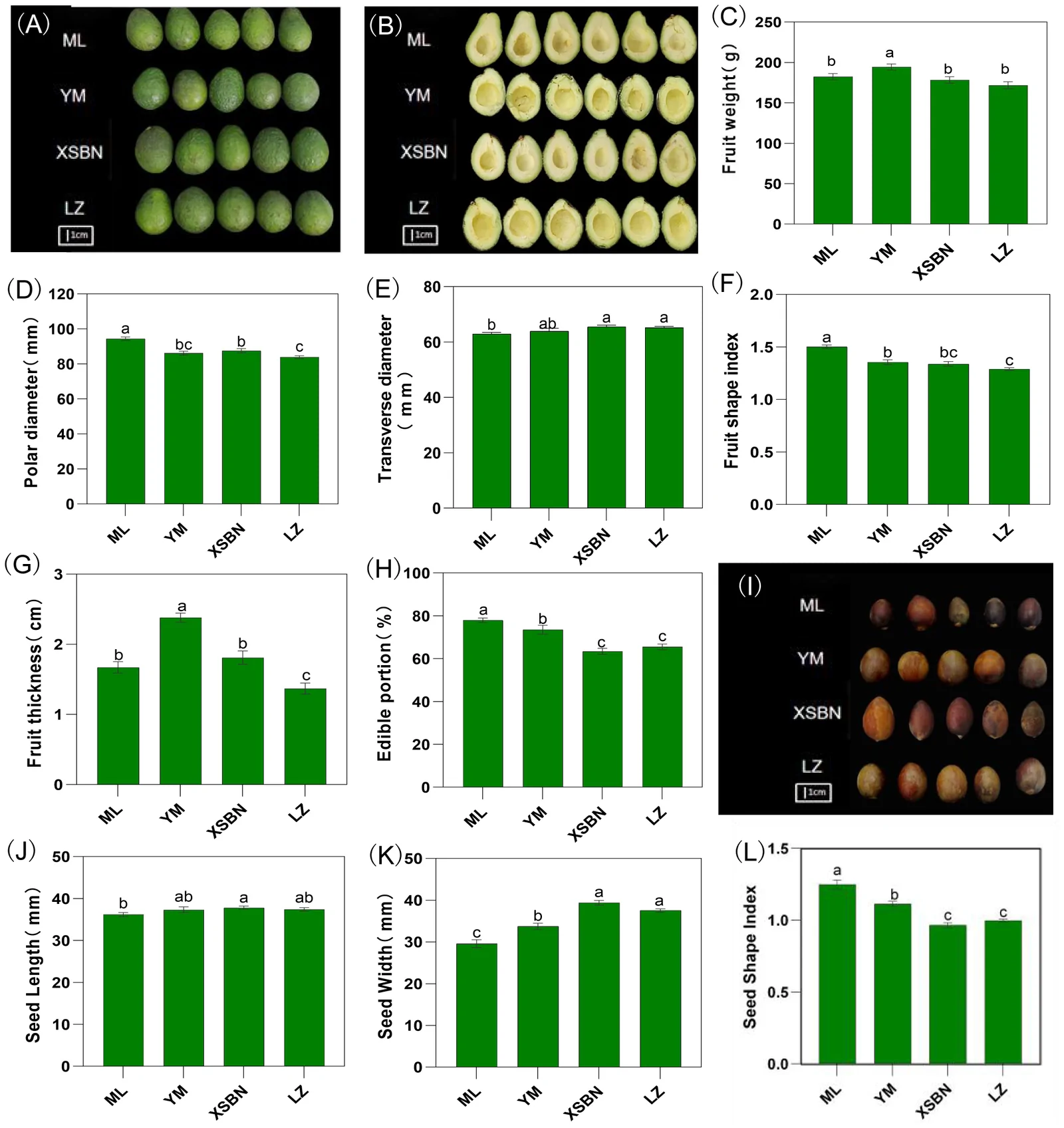
The appearance quality of avocado fruits from different regions. **(A)** appearance of avocado fruits from different production regions (bar = 1 cm), **(B)** longitudinal section morphology of fruits (bar = 1 cm), **(C)** fruit weight, **(D)** polar diameter (fruit longitudinal diameter), **(E)** transverse diameter, **(F)** fruit shape index, **(G)** fruit thickness (pulp thickness), **(H)** edible portion (edible portion yield), **(I)** seed appearance (bar = 1 cm), **(J)** seed length (seed longitudinal diameter); **(K)** seed width (seed transverse diameter), **(L)** seed shape index. The results shown are the means ± SDs (*n*=3). Different letters indicate significant differences (*P* < 0.05 according to Tukey’s test).

The appearance and morphology of fruit is an important basis for evaluating its commodity value. Significant regional differences were observed in fruit physical properties (**Figures 1A–C**). YM fruits exhibited the largest single fruit weight and thickest pulp, while ML fruits were characterized by the greatest longitudinal diameter and a distinctly pear-shaped morphology (highest fruit shape index). In contrast, XSBN fruits showed the largest transverse diameter, and LZ fruits generally had smaller physical dimensions across most measured parameters (**Figures 1D–H**). These regional differences in fruit morphology are consistent with previous observations that environmental factors significantly affect avocado fruit development (Lahack et al., 2025). Regarding pulp characteristics, YM fruits had the greatest pulp thickness, which was consistent with their larger fruit weight and aligns with commercial preferences for high-yield varieties (Yangaza et al., 2024). ML fruits showed the highest edible portion yield, largely attributable to their smaller seed transverse diameter and more slender seed shape (**Figures 1I–L**).

It is important to note that the four production areas differ substantially in climatic conditions (**Supplementary Tables 1**, **3**). YM has the highest mean annual temperature (23.1 °C) and longest sunshine duration (2183.8 h) but the lowest precipitation (700 mm); ML has the lowest temperature (19.6 °C) and highest altitude (1198.53 m); XSBN has moderate temperature and precipitation; and LZ has the shortest sunshine duration (1555 h). These climatic differences likely contribute to the observed variations in fruit morphology and quality, alongside soil properties and management practices. However, due to the observational nature of this study with only one orchard per region, the individual effects of climate, soil, and management cannot be statistically separated. The associations between soil properties and fruit quality reported in this study should therefore be interpreted with the understanding that climatic factors may also contribute to the observed differences.

Collectively, these results demonstrate that Hass avocado physical properties vary significantly across production regions. YM fruits are characterized by large size and thick pulp (high-yield potential), ML fruits exhibit a distinctive pear shape and high edible yield (consumer-preferred quality traits), while XSBN and LZ fruits show intermediate or smaller physical dimensions. These patterns are consistent with previous findings that morphological traits, while heritable, are modulated by environmental factors (Li et al., 2024; Lahack et al., 2025).

### 3.2 Fruit nutritional quality from different avocado producing areas and their analysis

**Figure 2:**
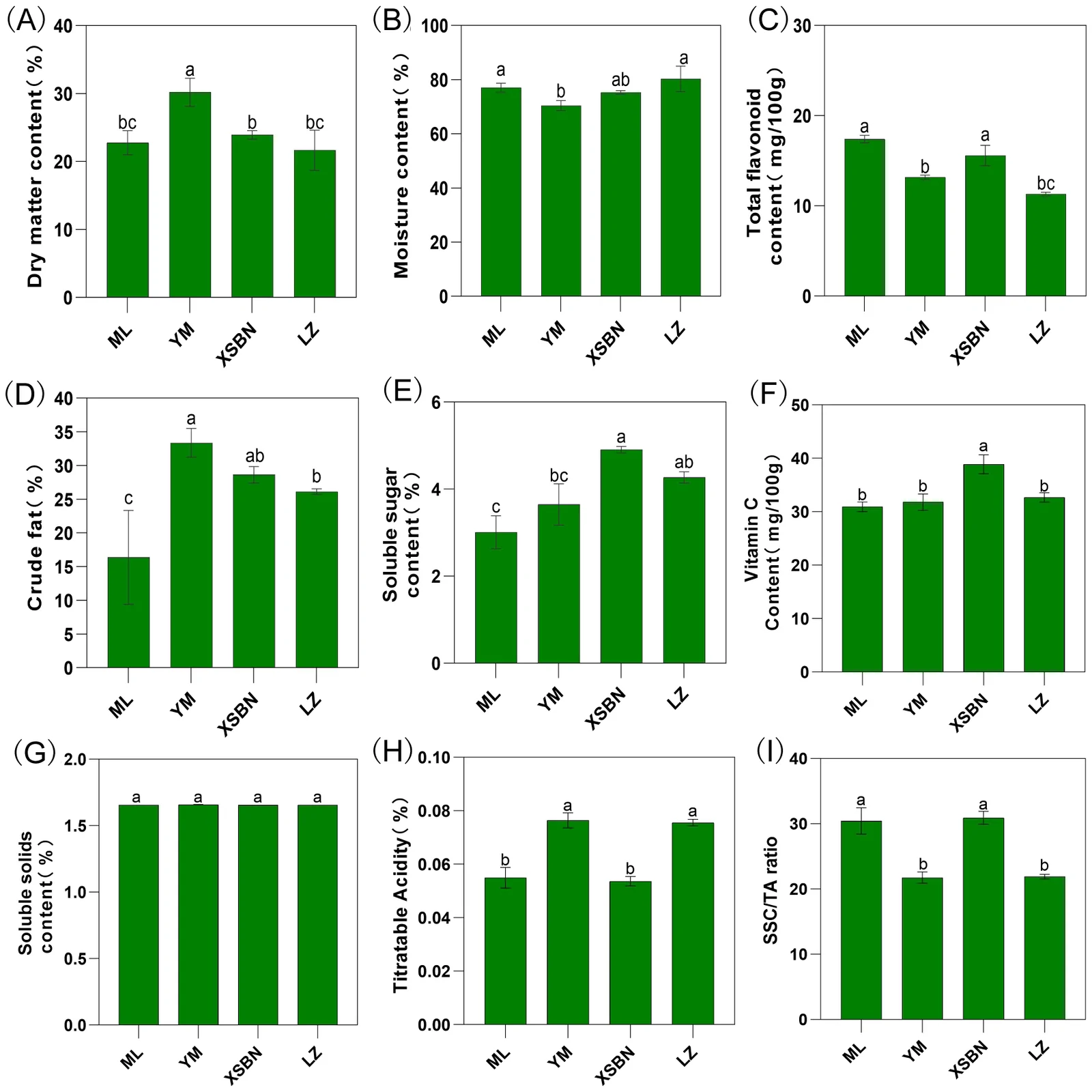
The nutritional quality of avocados from different regions. **(A)** dry matter content, **(B)** moisture content, **(C)** total flavonoid content, **(D)** crude fat, **(E)** soluble sugar, **(F)** vitamin C content, **(G)** soluble solid content, **(H)** titratable acidity, **(I)** SSC/TA ratio. The results are presented as the means ± SDs (*n*=3). Different letters indicate significant differences (*P* < 0.05 according to Tukey’s test).

The nutritional quality indices provide an important reference for evaluating avocado quality across different regions. Avocado has high nutritional value, containing three macronutrients including lipid, protein, and carbohydrate, and rich in Cu and Mn (Reddy et al., 2014). Significant regional differences were observed in nutritional quality parameters (**Figure 2**). YM fruits exhibited the highest dry matter content, crude fat content, and titratable acidity, but the lowest water content (**Figures 2A, B, D, H**). The crude fat content of YM avocado was notably high, which is consistent with the positive correlation between dry matter accumulation and oil synthesis in avocado fruits, as oil is the main component of avocado dry matter (Dreher and Davenport, 2013). XSBN fruits excelled in soluble sugar content, vitamin C content, and SSC/TA ratio (**Figures 2E, F, I**). ML fruits showed the highest total flavonoid content, while LZ fruits generally had lower values across most nutritional parameters (**Figure 2C**). No significant differences were observed in soluble solids content among the four regions (**Figure 2G**). The regional differences in nutritional composition highlight the importance of geographical indications for avocado (Méndez Hernández et al., 2024; López-Hoyos et al., 2024). López-Hoyos et al. (2024) also pointed out that soil characteristics, climate, and harvest seasonality are decisive factors in the multidimensional quality of Hass avocados in tropical regions, which further confirms the view that regional environmental conditions can form unique quality characteristics.

Our results of this study confirm the previous findings that the nutritional components of avocado vary significantly with different regions (Méndez Hernández et al., 2024). Fruit minerals play an important role in the quality of Hass avocado. The quality can be improved by optimizing the concentration of mineral elements, such as increasing calcium absorption to increase single fruit weight (Hofman et al., 2002).

Collectively, YM avocado performed significantly better in crude fat, dry matter content, and titratable acid, indicating higher nutritional density. ML avocado had relatively high total flavonoid content, which is noteworthy given the recognized antioxidant properties of flavonoids (Viera et al., 2023). XSBN avocado was outstanding in soluble sugar, SSC/TA ratio, vitamin C, and micronutrients such as Fe, Zn, and P, making it suitable as a supplementary source of these elements. LZ fruits showed comparatively lower values for most nutritional parameters. These distinct regional nutritional profiles demonstrate that it is feasible to select avocado based on target nutrients, indicating that quality characteristics have distinct regional signatures that can guide variety selection for specific production areas.

### 3.3 Fruit mineral elements from different avocado producing areas

**Figure 3:**
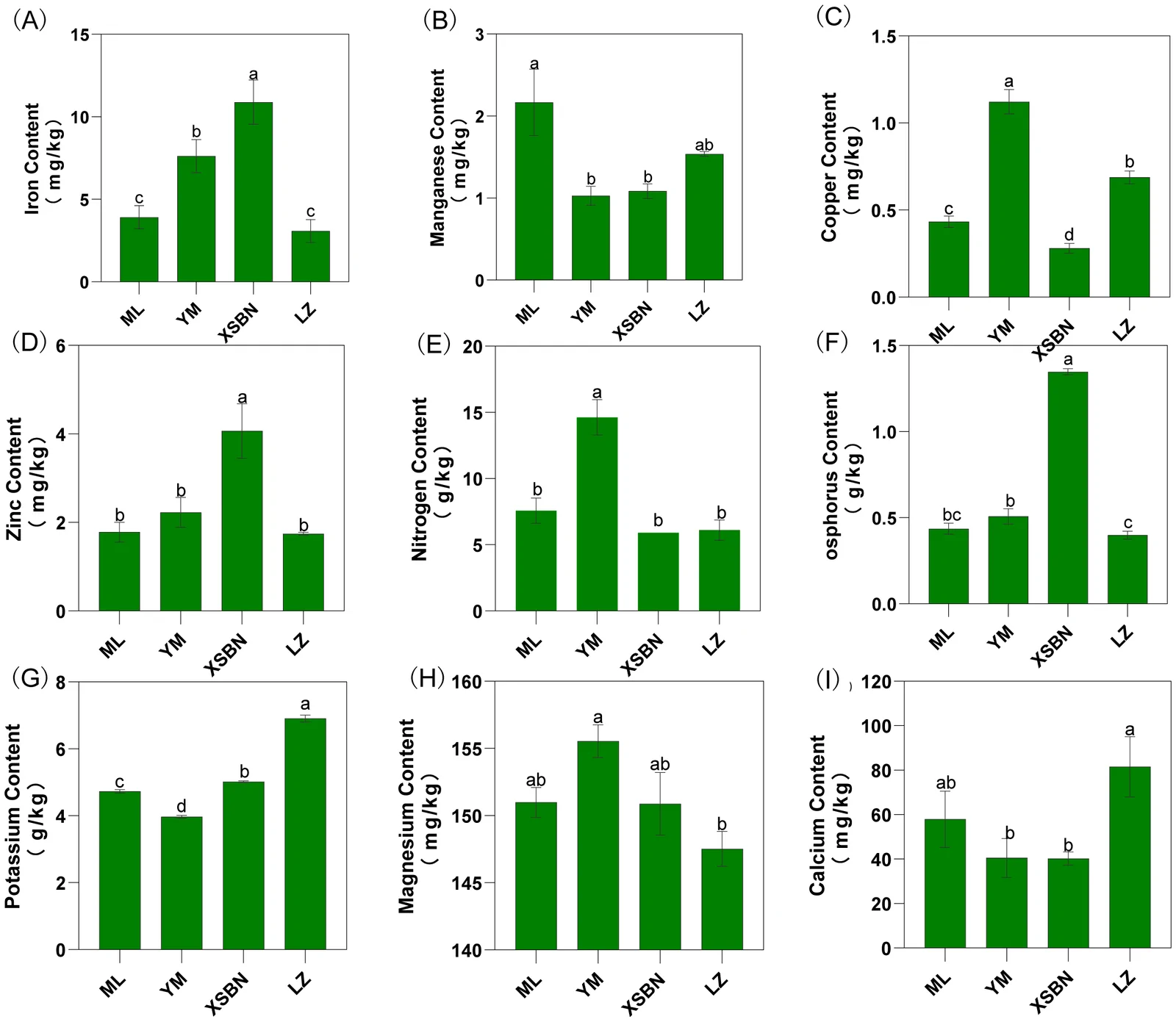
The accumulation differences of mineral elements in avocado fruits from different production areas. The concentrations of **(A)** Fe, **(B)** Mn, **(C)** Cu, **(D)** Zn, **(E)** N, **(F)** P, **(G)** K, **(H)** Mg, and **(I)** Ca were measured. The results are presented as the means ± SDs (*n*=3). Different letters indicate significant differences (*P* < 0.05 according to Tukey’s test).

Avocado fruits are highly nutritious and rich in various mineral elements. Significant regional differences were observed in the accumulation of mineral elements (**Figure 3**). XSBN fruits showed the highest concentrations of Fe, Zn, and P, and were among the highest for Mn (**Figures 3A, D, F**). ML fruits exhibited the highest Mn content, with moderate levels of other elements (**Figure 3B**). YM fruits had the highest Cu, N, and Mg contents, while LZ fruits were characterized by the highest K and Ca contents but generally lower levels of other mineral elements (**Figures 3C, E, G-I**).

Notably, YM fruits showed the highest N content, which was consistent with their larger fruit size and thicker pulp, suggesting that nitrogen availability may contribute to vegetative and fruit growth. LZ fruits exhibited the highest K and Ca concentrations, which may influence fruit quality and postharvest performance (Hofman et al., 2002). These results confirm previous findings that avocado nutritional composition varies substantially with growing region (Ullah and Joyce, 2024). These regional differences in mineral element composition, combined with the nutritional quality patterns described above, demonstrate that Hass avocados from different production areas possess distinct and characteristic mineral profiles that may influence both nutritional value and functional properties.

### 3.4 Rhizosphere soil nutrients from different avocado producing areas

**Figure 4:**
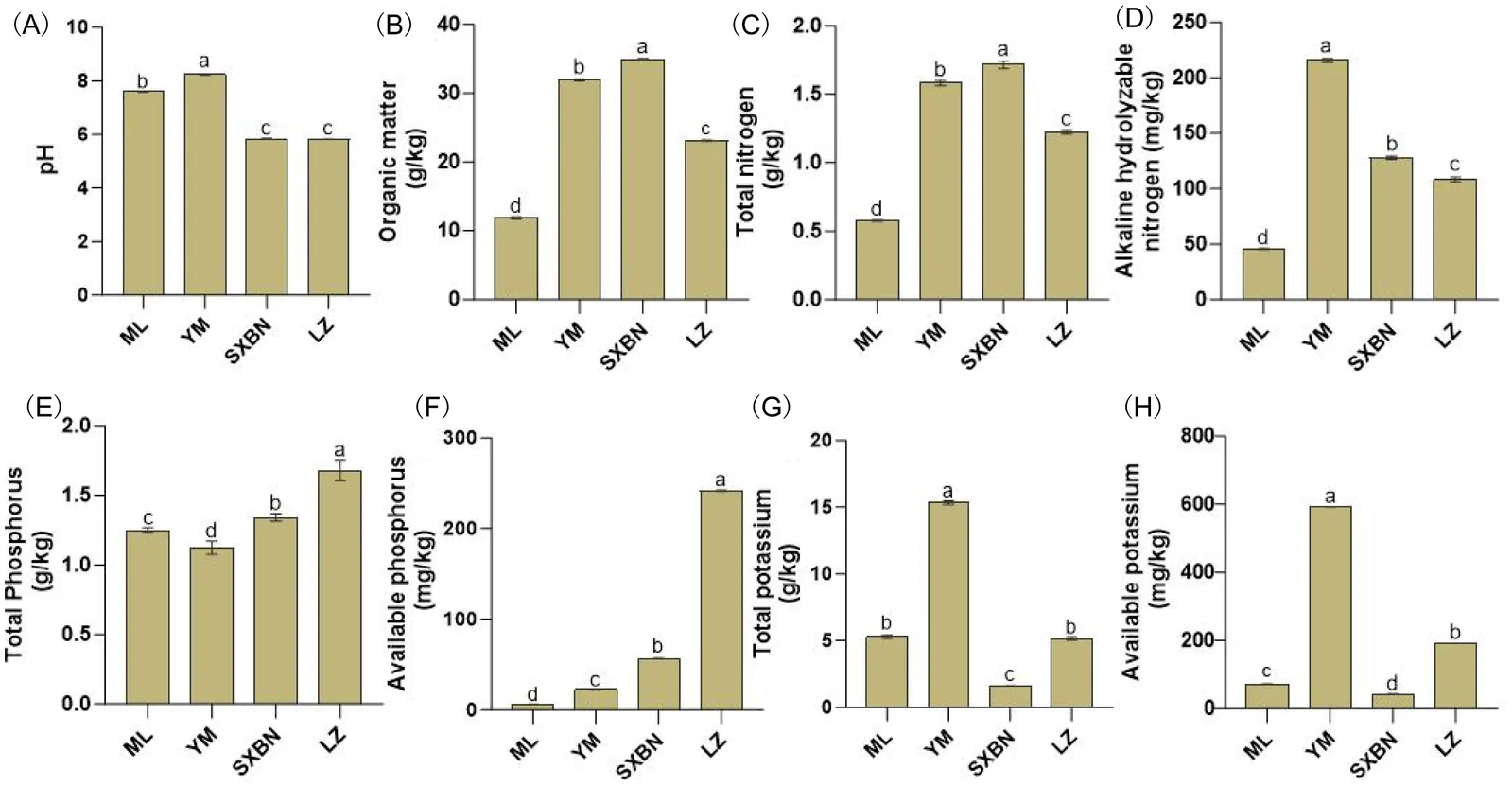
Nutrient differences in the rhizosphere soil of avocados from different production areas. **(A)** pH, **(B)** organic matter content, **(C)** total nitrogen, **(D)** alkaline hydrolyzable nitrogen, **(E)** total phosphorus, **(F)** available phosphorus, **(G)** total potassium, **(H)** available potassium. The results are presented as the means ± SDs (*n*=3). Different letters indicate significant differences (*P* < 0.05 according to Tukey’s test).

The rhizosphere soil acts as a critical interface between fruit trees and the external environment, directly determining tree health and economic benefits through its ecological balance. Analysis revealed that soil pH in the YM rhizosphere was 8.24, significantly higher than in ML, XSBN, and LZ, particularly 41.63% higher than LZ (**Figure 4A**). Soil organic matter content was significantly highest in XSBN (34.98 g/kg), being 194.60% higher than ML (**Figure 4B**). Total nitrogen content was significantly highest in XSBN (1.72 g/kg), notably 196.83% higher than ML (**Figure 4C**). Alkali-hydrolyzable nitrogen was significantly highest in YM (215.64 mg/kg), exceeding that in XSBN, LZ, and ML by 370.31% compared to ML (**Figure 4D**).

Total phosphorus was significantly highest in LZ (1.68 g/kg), being 49.46% higher than YM specifically (**Figure 4E**). Available phosphorus in LZ was 241.72 mg/kg, higher than in XSBN and YM, and significantly higher than in ML (**Figure 4F**). Total potassium was significantly highest in YM (15.33 g/kg) compared to LZ and ML, and significantly higher than in XSBN (**Figure 4G**). Available potassium was significantly highest in YM (592.05 mg/kg), particularly 1259.81% higher than in XSBN (**Figure 4H**). These soil parameter variations across orchards are more pronounced than those reported in previous comparative studies (González-Vences et al., 2024; Ullah and Joyce, 2024), possibly due to differences in parent materials and management practices.

### 3.5 Rhizosphere soil mineral elements from different avocado producing areas

As presented in **Table 1**, soil calcium content was significantly highest in YM (13.80 g/kg), notably 5646.75% higher than in XSBN. Mg content was highest in ML (1.77 g/kg), being 416.82% higher than in LZ. Iron content was significantly highest in ML (96.56 g/kg), exceeding that in XSBN, LZ, and YM by 180.90% compared to YM. Manganese content was significantly highest in ML (1.47 g/kg), being 235.69% higher than in YM. Copper content was significantly highest in ML (119.50 g/kg), notably 347.25% higher than in YM. Zinc content was significantly highest in LZ (152.99 g/kg), being 189.15% higher than in YM. Cadmium content was highest in ML (0.63 g/kg), particularly 1084.84% higher than in XSBN. Arsenic content was highest in LZ (44.58 g/kg), being 873.10% higher than in XSBN (**Table 1**).

**Table 1:** The differences in mineral elements in the rhizosphere soil among different avocado production areas.

Planting area	Ca g/kg	Mg g/kg	Fe mg/kg	Mn mg/kg	Cu mg/kg	Zn mg/kg	Cd mg/kg	As mg/kg
ML	2.14 ± 0.013 b	1.77 ± 0.004 a	96.56 ± 0.173 a	1.47 ± 0.005 a	119.50 ± 0.712 a	128.71 ± 0.215 b	0.63 ± 0.003a	11.75 ± 0.064 c
YM	13.81 ± 0.003a	0.51 ± 0.002 c	34.38 ± 0.055 d	0.44 ± 0.008 c	26.72 ± 0.251 d	52.91 ± 0.198 d	0.07 ± 0.001 c	12.11 ± 0.095 b
XSBN	0.24 ± 0.001 d	0.80 ± 0.002 b	92.83 ± 0.267 b	1.29 ± 0.004 b	102.97 ± 0.362 b	64.48 ± 0.374 c	0.05 ± 0.001 d	4.58 ± 0.078 d
LZ	0.38 ± 0.003 c	0.34 ± 0.001 d	76.62 ± 0.161 c	0.25 ± 0.003 d	36.84 ± 0.205 c	152.99 ± 0.355 a	0.29 ± 0.002 b	44.58 ± 0.020 a

The results are presented as the means ± SDs (n=3 biological replicates per region, where each replicate represents one tree). Different lowercase letters in the same row indicate significant differences among regions (one-way ANOVA, P < 0.05, followed by Tukey's multiple range test; all reported differences had FDR-adjusted q < 0.05). Ca, calcium; Mg, magnesium; Fe, iron; Mn, manganese; Cu, copper; Zn, zinc; Cd, cadmium; As, arsenic. ML, Menglian; YM, Yuanmou; XSBN, Xishuangbanna; LZ, Longzhou.

### 3.6 Rhizosphere soil enzymes from different avocado producing areas

**Figure 5:**
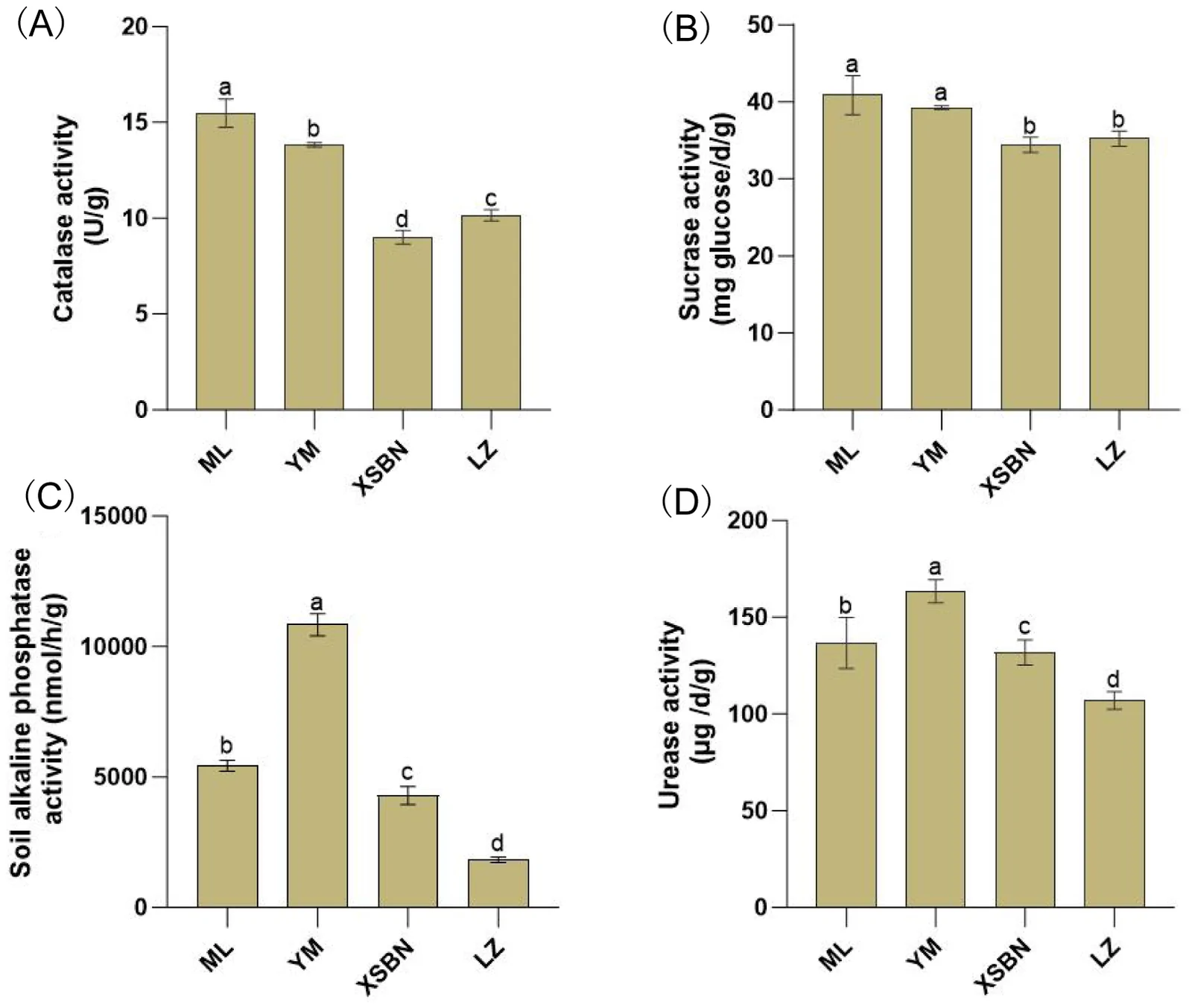
The differences in rhizosphere soil enzyme activities among different avocado production areas. **(A)** catalase activity, **(B)** sucrase activity, **(C)** soil alkaline phosphatase activity, **(D)** urease activity. The results are presented as the means ± SDs (*n*=3). Different letters indicate significant differences (*P* < 0.05 according to Tukey’s test).

Measurements of CAT, sucrase, alkaline phosphatase, and urease activities in the rhizosphere soil revealed that CAT activity was significantly highest in ML (15.49 U/g), being 71.93% higher than in LZ (**Figure 5A**). Sucrase activity in ML and YM (40.91 U/g and 39.25 U/g, respectively) was significantly higher than in XSBN and LZ, with ML being approximately 18.82% higher than XSBN (**Figure 5B**). Alkaline phosphatase activity was significantly highest in YM (10838.82 U/g), exceeding that in XSBN, LZ, and ML by 152.70%, 492.24%, and 99.43%, respectively (**Figure 5C**). Urease activity was significantly highest in YM (163.60 U/g), being 24.11%, 52.95%, and 19.66% higher than in XSBN, LZ, and ML, respectively (**Figure 5D**). The highest sucrase and CAT activities, along with elevated levels of multiple mineral elements in the ML orchard soil, suggest a capacity to provide ample energy for avocado cultivation.

Soil physicochemical properties significantly affect plant growth (Yang et al., 2025). Comparative analysis of soil properties across the different avocado producing areas revealed significant differences. ML soil contained higher Fe, Mn, Cu, Mg and Cd concentrations, and exhibited stronger CAT and sucrase activities. The soil pH of YM soil was significantly higher, and the contents of alkali-hydrolyzable N, total K, available K and Ca, as well as the activities of alkaline phosphatase and urease were the highest. The contents of soil organic matter and total N in XSBN soil were the highest, while the contents of total P, available P, Zn and As in LZ soil were the highest. The differences in soil parameters among these orchards are more significant than those reported in previous comparative studies (Sinsabaugh et al., 2008; Ullah and Joyce, 2024; Vida et al., 2016), which may be related to differences in soil parent materials and management measures.

The activities of CAT and sucrase in ML orchard soil were the highest, and the contents of various mineral elements were high, indicating that ML orchard soil has the potential to provide sufficient nutrients and energy for avocado cultivation. YM soil is characterized by high pH and organic matter content. The contents of alkali-hydrolyzable nitrogen, total K and available K are the highest, but the content of P is relatively low. It may be necessary to supplement organic fertilizer, especially phosphorus fertilizer, in cultivation. This result is consistent with the similar nutrient imbalance phenomenon found in Mexican orchards through nutritional diagnosis (González-Vences et al., 2024). Although the content of organic matter and total N in XSBN soil is high, the content of available P is low and the content of K is the lowest. Therefore, targeted fertilization strategies focusing on P and K are necessary. Regarding heavy metals, soil As content in LZ (44.58 mg/kg) exceeded the Chinese risk screening value for agricultural soil (20 mg/kg), while soil Cd in ML (0.63 mg/kg) was close to the screening threshold (0.6 mg/kg). These soil test results can be regarded as a preventive finding. Further monitoring is still necessary to assess the food safety issues.

### 3.7 Correlation analysis among fruit quality traits

**Figure 6:**
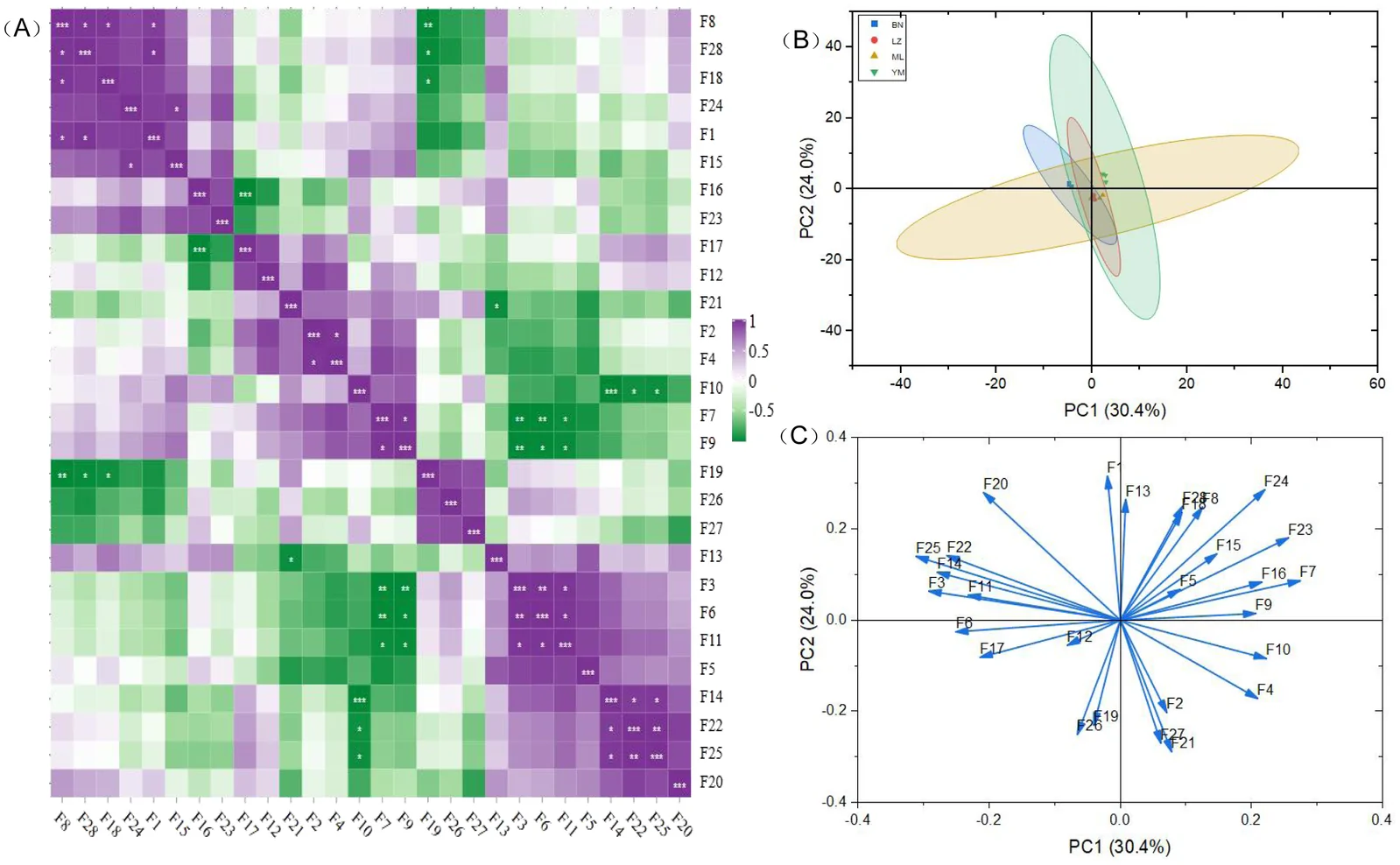
Analysis of the interaction between avocado Fruit quality. **(A)** Correlation heatmap of fruit quality indicators, **(B)** principal component analysis (PCA) score plot of fruit quality traits from different production areas, **(C)** PCA loading plot showing the contribution of each quality parameter to the principal components. F1: Fruit Weight, F2: Fruit Longitudinal Diameter, F3: Fruit Transverse Diameter, F4: Fruit Shape Index, F5: Seed Longitudinal Diameter, F6: Seed Transverse Diameter, F7: Seed Shape Index, F8: Pulp Thickness, F9: Edible Portion Yield, F10: Soluble Protein Content, F11: Soluble Sugar Content, F12: Total Flavonoid Content, F13: Crude Fat Content, F14: Vitamin C Content, F15: Soluble Solids Content (SSC), F16: Titratable Acidity, F17: Soluble Solids to Titratable Acidity Ratio (SSC/TA Ratio), F18: Dry Matter Content, F19: Water Content, F20: Iron (Fe) Content, F21: Mn Content, F22: Zn Content, F23: Cu Content, F24: Total N Content, F25: Total P Content, F26: Total K Content, F27: Ca Content, F28: Mg Content. The results are expressed as correlation coefficients or PCA loadings, and asterisks indicate significant correlations at different probability levels (*P < 0.05, **P < 0.01, ***P < 0.001).

Correlation analysis of avocado fruit quality indicators identified several significant relationships that collectively illustrate the integrated nature of fruit quality formation (**Figure 6A**). Fruit weight, pulp thickness, and dry matter content were positively intercorrelated, while water content showed negative associations with these parameters, suggesting a coordinated accumulation pattern where assimilates are allocated towards increasing fruit size and dry matter storage, with Mg potentially playing a role in carbohydrate transport (Cakmak and Yazici, 2010).

Several antagonistic relationships were also observed. Soluble protein content was negatively correlated with vitamin C, Zn, and P, while vitamin C showed positive correlations with Zn and P. This reciprocal pattern may reflect a metabolic trade-off in carbon allocation between protein synthesis and ascorbic acid biosynthesis, with Zn and P functioning synergistically with vitamin C in the fruit’s antioxidant network (Smirnoff and Wheeler, 2000; Bhuyan et al., 2019; Hänsch and Mendel, 2009). Crude fat content was negatively correlated with Mn content, consistent with the hypothesis that excessive manganese may interfere with lipid biosynthesis (Hänsch and Mendel, 2009; Millaleo et al., 2010), though this interpretation requires experimental validation.

Principal Component Analysis (PCA) of multiple quality traits yielded two principal components, PC1 (30.4% variance) and PC2 (24.0% variance), which effectively distinguished the four regions (**Figures 6B, C**). PC1, primarily defined by fruit shape index, seed shape index, edible portion yield, soluble protein, and titratable acidity, represented external morphology and nutritional quality. PC2, mainly defined by fruit weight, Fe, and total N, reflected physical properties and mineral composition. The PCA biplot clearly separated ML (high edible yield and flavonoids) from YM (high fruit weight and fat content), while XSBN and LZ occupied intermediate positions. This differentiation provides an exploratory basis for region-specific breeding strategies (Méndez Hernández et al., 2024; López-Hoyos et al., 2024). However, these regional differences may be influenced by a variety of confounding factors, including climate, management practices, and soil characteristics, and these factors need to be further separated in future research.

### 3.8 Interplay between fruit quality and rhizosphere soil characteristics

To understand associations between fruit quality and soil properties, we examined correlations across three functional dimensions: soil enzymes mediating nutrient bioavailability, mineral elements involved in plant metabolism, and potentially toxic elements that may exert stress effects. Interpretations are presented at three levels: statistical correlations observed, hypotheses derived from prior literature, and experimentally demonstrated mechanisms.

**Figure 7:**
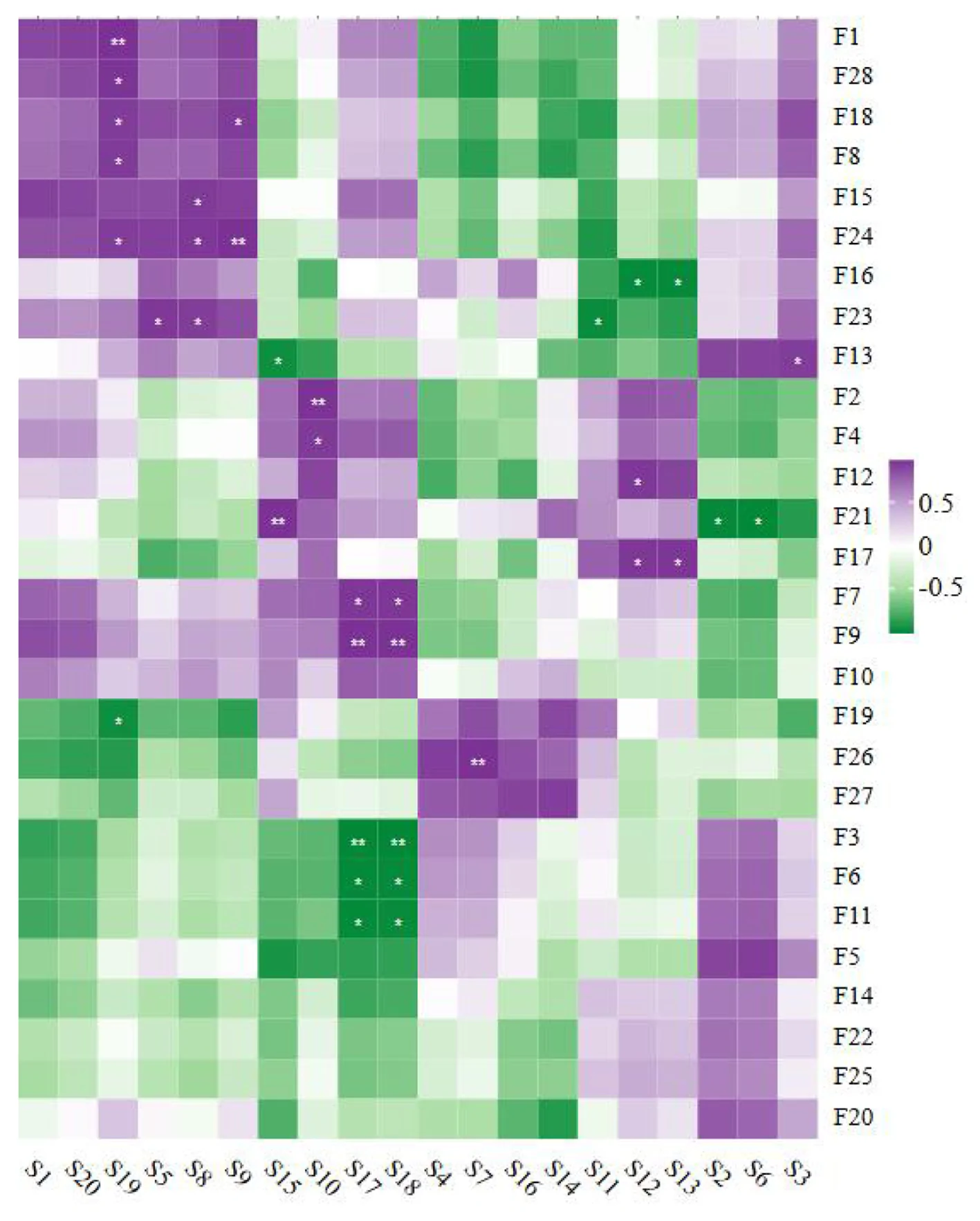
Analysis of the Interaction between avocado fruit quality and rhizosphere soil characteristics. Correlation heatmap between fruit quality indicators and soil physicochemical properties, correlation heatmap between fruit quality indicators and soil enzyme activities. Fruit Quality Traits (F1-F28): F1: Fruit Weight, F2: Fruit Longitudinal Diameter, F3: Fruit Transverse Diameter, F4: Fruit Shape Index, F5: Seed Longitudinal Diameter, F6: Seed Transverse Diameter, F7: Seed Shape Index, F8: Pulp Thickness, F9: Edible Portion Yield, F10: Soluble Protein Content, F11: Soluble Sugar Content, F12: Total Flavonoid Content, F13: Crude Fat Content, F14: Vitamin C Content, F15: Soluble Solids Content (SSC), F16: Titratable Acidity, F17: Soluble Solids to Titratable Acidity Ratio (SSC/TA Ratio), F18: Dry Matter Content, F19: Water Content, F20: Fe Content, F21: Mn Content, F22: Zn Content, F23: Cu Content, F24: Total N Content, F25: Total P Content, F26: Total K Content, F27: Ca Content, F28: Mg Content. Soil Properties (S1-S20): S1: Soil pH, S2: Soil Organic Matter (SOM) Content, S3: Soil Total N Content, S4: Soil Total P Content, S5: Soil Total K Content, S6: Soil Alkali-hydrolyzable N, S7: Soil Available P, S8: Soil Available K, S9: Soil Ca Content, S10: Soil Mg Content, S11: Soil Fe Content, S12: Soil Mn Content, S13: Soil Cu Content, S14: Soil Zn Content, S15: Soil Cd Content, S16: Soil As Content, S17: Soil Catalase Activity, S18: Soil Sucrase Activity, S19: Soil Alkaline Phosphatase Activity, S20: Soil Urease Activity. The results are expressed as correlation coefficients, and asterisks indicate significant correlations at different probability levels (*P < 0.05, **P < 0.01, ***P < 0.001).

Soil enzymes as drivers of nutrient mobilization. The most prominent associations were observed with alkaline phosphatase (ALP). Soil ALP activity correlated positively with fruit weight (P < 0.01), pulp thickness, dry matter content, fruit total N, and Mg content (all P < 0.05), and negatively with water content (**Figure 7**). This associative pattern suggests that ALP-mediated P mineralization may be linked to ATP synthesis and energy-dependent processes (Sinsabaugh et al., 2008). The concurrent increase in fruit Mg with ALP activity is consistent with Mg-ATP as the substrate for numerous kinases, suggesting a potential P-Mg synergy at the soil-plant interface (Cakmak and Yazici, 2010). However, this hypothesized cascade requires experimental testing. In contrast, soil CAT and sucrase activities correlated negatively with fruit transverse diameter, seed transverse diameter, and soluble sugar content (P < 0.05) (**Figure 7**). A plausible hypothesis is that high sucrase activity may accelerate sucrose hydrolysis in soil, fueling microbial competition with roots for carbon and potentially reducing photosynthate allocation to fruit development (Sinsabaugh et al., 2008; Kuzyakov and Xu, 2013). This hypothesis requires targeted investigation.

Mineral elements as modulators of fruit morphology and secondary metabolism. Soil Mg content showed the strongest positive correlations with fruit longitudinal diameter (P < 0.01) and fruit shape index (P < 0.05). This extends Mg’s known roles beyond chlorophyll synthesis, suggesting its potential involvement in cell wall biosynthesis and phloem loading (Cakmak and Yazici, 2010; Hermans et al., 2013). Soil Mn content correlated positively with fruit total flavonoids (P < 0.05) and the SSC/TA ratio (P < 0.05), consistent with Mn’s role as a cofactor for phenylalanine ammonia-lyase (PAL) in flavonoid biosynthesis (Kováčik et al., 2010; Tsednee, 2025). This provides an associative rationale for the high flavonoid content in ML avocados, which also had the highest soil Mn. Soil Ca content correlated positively with fruit dry matter and total N (P < 0.05) (**Figure 7**), consistent with Ca’s role in maintaining membrane integrity and nitrogen assimilation (Bonomelli et al., 2019). However, these mechanistic interpretations require validation through future experimental research.

Heavy metals as potential stress factors. A negative correlation was observed between soil Cd content and fruit crude fat and Mn contents. A plausible hypothesis, derived from plant stress physiology, is that Cd-induced oxidative stress may disrupt lipid biosynthesis (Hänsch and Mendel, 2009; Gallego et al., 2012), though this was not tested directly. The important point is that the As content in the soil of the LZ area exceeds the risk screening value in China (20 mg/kg), while the Cd content in the soil of the ML area is close to the screening threshold (0.6 mg/kg). These soil observation results should be regarded as reference for routine soil monitoring and further investigation, rather than evidence of food safety risks. This study did not conduct human exposure assessment or calculate the bioaccumulation index. The observed correlation here may also be influenced by other regional factors that were not controlled in the design of this study.

### 3.9 Study limitations, practical implications, and future perspectives

While this study provides valuable empirical links between soil properties and avocado fruit quality, several critical limitations must be acknowledged. First and foremost, agricultural management practices, including irrigation methods, fertilization regimes, and additional orchard practices, were not incorporated into the statistical analyses. As shown in **Supplementary Table 2**, the four orchards differ substantially in management: ML uses drip irrigation with mulching; YM uses flood irrigation with higher water application due to high temperatures; XSBN uses sprinkler irrigation with supplementary irrigation only during dry spells; and LZ uses drip irrigation with periodic liming due to acidic soil. Fertilizer application rates (1.5 kg/tree per application) and frequencies (4 times/year) are similar across regions, but irrigation methods and additional practices (mulching, liming) vary considerably. These management differences could substantially explain variations in both soil characteristics and fruit quality. Because management practices covary with soil properties and climate across regions, it is impossible to statistically separate their individual effects in this observational study design.

Second, a fundamental limitation of this study is that climatic variables (temperature, precipitation, and sunshine duration) were not incorporated into the statistical analyses. As shown in **Supplementary Tables 1** and **3**, the four regions differ substantially in mean annual temperature (ranging from 19.6 °C to 23.1 °C), annual precipitation (ranging from 700 mm to 1500 mm), and sunshine duration (ranging from 1555 h to 2183.8 h). These climatic factors are well-documented to influence avocado fruit development, quality, and yield (López-Hoyos et al., 2024; Yangaza et al., 2024). However, because only one orchard was selected per region in this observational study, climate, soil properties, and management practices are completely confounded—they all vary simultaneously across regions. Therefore, it is impossible to statistically separate the individual contributions of soil effects from climatic effects. This is a limitation of the study design that cannot be resolved by alternative statistical methods or by increasing sample size.

Third, only one orchard was selected for each production area, and multiple environmental and management factors (climate, irrigation, fertilization, altitude, tree age, and rootstock characteristics) were not controlled among the different areas. Although these four orchards represent the typical situations of their respective production areas, they still have differences in multiple aspects. Therefore, the observed differences in fruit quality cannot be entirely attributed to soil properties. This is a fundamental limitation of the study design. Thus, our results should be interpreted as associations that generate hypotheses, not as evidence of causation. The manuscript should therefore be viewed as an observational comparative study of representative orchards, with all findings presented as associative patterns rather than causal relationships. The fruit quality characteristics measured in these representative orchards can reflect the typical fruit quality attributes of Hass avocados grown in these four production areas to a certain extent, but the underlying causes of these differences remain to be determined. Furthermore, a key limitation of this study lies in the fact that all the mechanistic explanations proposed by the research are based on correlation analysis and existing literature, rather than direct experimental evidence generated by this study. These explanations should be regarded as having significant guiding significance for future research.

Fourth, the single-season sampling and limited the sample size (n=4 regions) restrict our ability to assess temporal stability and to apply advanced multivariate models (e.g., random forest, structural equation modeling) that could quantify the relative contributions of individual factors. Furthermore, the small number of regions (n=4) limits the statistical power to disentangle the effects of individual soil factors from regional confounding variables. It should also be noted that the three trees sampled within each region were clustered within three locations, and while we treated the tree as the experimental unit, the spatial clustering within orchards may introduce some degree of non-independence.

Despite these constraints, our findings offer exploratory, hypothesis-generating insights for regional soil management. In YM, where ALP activity is highest and fruit weight and fat content are superior, maintaining P availability through ALP stimulation (e.g., by organic matter amendment) may be worth investigating. In ML, where soil Mg and Mn are high and correlate with high edible yield and flavonoid content, ensuring adequate Mg and Mn supply may be associated with these desirable traits. Regarding heavy metals, it must be acknowledged that there are several significant limitations. The As content in the soil of the LZ area exceeds the risk screening value for agricultural soil in China, while the Cd content in the soil of the ML area is close to the screening threshold. Therefore, the increase in heavy metal concentrations in the soil should be regarded as a problem that needs to be monitored in terms of soil quality. Although the negative correlation between soil cadmium and fruit quality traits is statistically significant, it may be influenced by other confounding factors and further research is needed to explore this in detail in order to clearly assess the absorption, transport, and accumulation of heavy metals by avocado trees. The high soil As in LZ and Cd in ML, although not yet translocated to fruit, necessitate proactive monitoring and the adoption of remediation strategies such as phosphate application (to compete with As uptake) or the selection of low-accumulating rootstocks (Zhao et al., 2022). These region-specific recommendations represent preliminary steps towards precision soil management for optimized avocado quality (Silber et al., 2018; Bonomelli et al., 2019).

Given these limitations, future research should adopt a multi-orchard, multi-season design with standardized management practices and simultaneous measurement of climatic variables to distinguish soil effects from climatic and agronomic factors. Specifically, future studies should: (i) include multiple representative orchards within each region that share similar management practices to allow statistical separation of regional (climate + soil) effects from management effects; (ii) design controlled experiments where management practices are systematically varied while soil and climate are held constant, to establish causal relationships between specific management practices and fruit quality; (iii) incorporate management variables directly into multivariate statistical models alongside soil and climatic variables to quantify their relative contributions; (iv) conduct controlled experiments under regulated environments where temperature, water availability, and nutrient supply can be manipulated independently; and (v) conduct multi-season sampling across multiple years to assess temporal stability of the observed associations.

Ideally, future research should include multiple representative orchards in each region to separate the regional effects from the orchard-specific effects. Specifically, controlled experiments should be designed to directly test the mechanistic hypotheses proposed in this study: (i) whether manganese availability affects lipid biosynthesis in avocado fruits; (ii) whether cadmium exposure inhibits acetyl-CoA carboxylase activity; (iii) whether magnesium supplementation enhances PAL activity and flavonoid accumulation; and (iv) whether increased sucrase activity in soil reflects competition between soil microorganisms and plant roots for carbon substrates. Future studies should also consider increasing the number of trees sampled per region to improve statistical power and generalizability. Controlled experiments, ideally under regulated environments, are essential to establish causal relationships between the key soil parameters identified here (ALP, Mg, Mn, Cd) and specific quality traits. Additionally, integrating soil microbial community analysis with enzyme activity measurements could further elucidate the ecological mechanisms governing nutrient cycling and carbon partitioning in the rhizosphere (Sinsabaugh et al., 2008; Kuzyakov and Xu, 2013), ultimately contributing to a more predictive framework for sustainable avocado cultivation. Furthermore, future studies with larger sample sizes and designed specifically for multiple comparison corrections would help validate the associations identified in this exploratory study.

## 4 Conclusion

This observational comparative study of representative orchards systematically characterized the regional variation in Hass avocado fruit quality across four Chinese production areas (ML, YM, XSBN, and LZ) and examined its associations with rhizosphere soil properties. The four representative orchards exhibited distinctive fruit quality profiles: YM fruits were characterized by the largest single fruit weight (194.36 g) and thickest pulp (2.38 cm), ML fruits showed the highest edible yield (77.91%) and a unique pear shape (fruit shape index 1.50), XSBN fruits excelled in vitamin C (38.87 mg/100 g) and soluble sugars (4.91%), and LZ fruits showed the highest K (6.91 mg/kg) and Ca (81.51 mg/kg) contents. Principal Component Analysis (PCA) effectively distinguished the four regions along two major axes. PC1 (30.4% variance) was primarily defined by fruit shape index, seed shape index, edible portion yield, soluble protein content, and titratable acidity, representing external morphology and nutritional quality attributes. PC2 (24.0% variance) was mainly defined by fruit weight, Fe content, and total nitrogen content, reflecting physical properties and mineral element composition. The PCA biplot clearly separated ML (high PC1, associated with high edible yield and flavonoid content) from YM (high PC2, associated with high fruit weight and crude fat content), while XSBN and LZ occupied intermediate positions with distinct nutritional profiles.

Correlation analyses revealed that soil properties showed differential associations with fruit quality traits across the three functional dimensions examined. Soil alkaline phosphatase activity showed the strongest positive correlations with fruit weight, pulp thickness, and dry matter content. Soil Mg content was positively associated with fruit longitudinal diameter and fruit shape index. Soil Mn content was positively correlated with fruit total flavonoid content. Conversely, soil CAT and sucrase activities showed negative correlations with fruit transverse diameter and soluble sugar content, and soil Cd content was negatively correlated with fruit crude fat content. The key soil variables that distinguished the four production regions were identified as follows: ML soils were characterized by the highest Fe, Mn, Cu, and CAT activity; YM soils by the highest pH, K, and alkaline phosphatase activity; XSBN soils by the highest organic matter and total N content; and LZ soils by the highest total P, available P, and As content. These regional soil characteristics coincided with the distinctive fruit quality attributes observed in each representative orchard.

In summary, these findings provide empirical evidence that Hass avocado fruit quality varies significantly across production regions in China and is associated with specific rhizosphere soil properties, particularly enzyme activities (alkaline phosphatase, CAT, sucrase) and mineral elements (Mg, Mn, Ca, Cd). The PCA and correlation analyses collectively indicate that soil factors, alongside climatic and management variables that were not separately controlled in this observational design, may be associated with the regional quality differentiation. It is important to emphasize that the substantial climatic differences among the four regions, including varying temperature (19.6–23.1 °C), precipitation (700–1500 mm), sunshine duration (1555–2183.8 h) and varying management practices, are completely confounded with soil properties in this study design, making it impossible to attribute the observed differences solely to soil characteristics. However, due to the observational and correlational nature of this study, and the confounding between regions and orchards (only one orchard per region), causal relationships cannot be inferred. The findings should be interpreted as generating hypotheses for future experimental validation rather than establishing causal effects. These findings offer an exploratory basis for generating hypotheses about region-specific soil management strategies, such as targeted fertilization and soil amendment practices, that could be tested in future controlled experiments to optimize avocado quality. Future studies should include multiple representative orchards within each region to separate regional effects from orchard-specific effects and to validate the associations observed here. The distinct regional quality profiles also highlight the potential for geographical indication and value-added differentiation of Chinese-grown Hass avocado.

## Data Availability

The original contributions presented in the study are included in the article/**Supplementary Material**. Further inquiries can be directed to the corresponding authors.
