# Supplementary material for: An observational comparative study of Hass avocado fruit quality and its associations with rhizosphere soil properties across four representative orchards in China
Source: Front Plant Sci. 2026 Aug 21;17:1890031. doi: 10.3389/fpls.2026.1890031 (PMC13541770; doi:10.3389/fpls.2026.1890031)
Supplement: Supplementary file 1 [file SupplementaryFile1.docx]

**Supplementary Table** **legends**

Table S1：Basic information of the four Hass avocado production areas

| Number of sample | Avocado base | Longiude | latitude | Altitude | Annual  average  temperature | Mean annual  precipitation | Average annua  lsunshine  duration |
| --- | --- | --- | --- | --- | --- | --- | --- |
| ML | Menglian | 99.5808° | 22.1226° | 1198.53 m | 19.6℃ | 1500 mm | 2050 h |
| YM | Yuanmou | 101.8753° | 25.8955° | 1090.69 m | 23.1℃ | 700 mm | 2183.8 h |
| XSBN | Xishuangbanna | 100.7768° | 21.8948° | 589.77 m | 21.7℃ | 1318.5 mm | 2060.5 h |
| LZ | Longzhou | 106.7709° | 22.2866° | 145.16 m | 22.4℃ | 1373.2 mm | 1555 h |

Note: The data in the table is compiled based on the climate information disclosed on the website of the county government of the planting area and the GPS positioning meteorological data of the planting area.

Table S2: Summary of irrigation and fertilization practices in the four avocado orchards

| Production area | Irrigation method | Irrigation frequency | Fertilizer type | Application rate (annual) | Fertilization frequency | Additional notes |
| --- | --- | --- | --- | --- | --- | --- |
| Menglian (ML) | Drip irrigation | Once every 7-10 days (dry season) | Compound fertilizer (N-P₂O₅-K₂O: 15-15- 15) + organic manure | 1.5 kg/tree per application + 10 kg/tree organic manure | 4 times/year (before flowering, fruit set, fruit expansion, post-harvest) | Mulching with crop residues |
| Yuanmou (YM) | Flood irrigation | Once every 10-15 days (dry season) | Compound fertilizer (N-P₂O₅-K₂O: 15-15- 15) + organic manure | 1.5 kg/tree per application + 10 kg/tree organic manure | 4 times/year (before flowering, fruit set, fruit expansion, post-harvest) | High temperature area, more irrigation water |
| Xishuangbanna (XSBN) | Sprinkler irrigation | Once every 5-7 days (dry season) | Compound fertilizer (N-P₂O₅-K₂O: 15-15- 15) + organic manure | 1.5 kg/tree per application + 10 kg/tree organic manure | 4 times/year (before flowering, fruit set, fruit expansion, post-harvest) | Supplementary irrigation only in dry spell |
| Longzhou (LZ) | Drip irrigation | Once every 7-10 days (dry season) | Compound fertilizer (N-P₂O₅-K₂O: 15-15- 15) + organic manure | 1.5 kg/tree per application + 10 kg/tree organic manure | 4 times/year (before flowering, fruit set, fruit expansion, post-harvest) | Acidic soil; periodic liming applied |

Note: The above information is based on field interviews conducted by the author's team with farmers and compiled from orchard management logs. Irrigation frequency rates vary slightly across different production areas due to climate conditions (rainfall, evaporation), soil type, and tree age (7-8 years).
